# Gap nonunion of forearm bones treated by modified Nicoll's technique

**DOI:** 10.4103/0019-5413.58611

**Published:** 2010

**Authors:** Dinesh K Gupta, Gaurav Kumar

**Affiliations:** Department of Orthopaedics, MLB Medical College, Jhansi, Uttar Pradesh, India; 1 Department of Orthopaedics, Jhansi Orthopaedic Hospital and Research Centre, Jhansi, Uttar Pradesh, India

**Keywords:** Gap nonunion of radius/ulna, intramedullary fixation, tricorticocancellous strut bone graft

## Abstract

**Background:**

The management of an atrophic nonunion with a gap following a fracture of the radius and/or ulna is a challenging problem. Various methods of treatment available in the literature are cortical tibial graft (Boyd), ulnar segment graft (Miller and Phalen), iliac crest graft (Spira), cancellous insert graft (Nicoll), vascularized fibular graft (Jupiter), and bone transport by ring fixator (Tesworth). The present study reports the results of tricorticocancellous bone block grafts using modified Nicoll's technique, in diaphyseal defects of forearm bones.

**Materials and Methods:**

A total of 38 forearm bones (either radius or ulna or both) in 23 patients with a gap of 1.5–7.5 cm were treated by debridement and tricorticocancellous bone block graft under compression with intramedullary nail fixation between June 1985 and June 2005. There were 15 male and 8 female patients. Sixteen patients had open and seven patients had closed fractures initially. Time of presentation since the original injury varied from 9 months to 84 months. Eighteen patients had already undergone one to three operations.

**Results:**

Thirty-six bones showed union at both host graft junctions. The mean duration of union was 17.5 weeks (range, 14–60 weeks). Two bones had union only at one host graft junction and did not show any evidence of callus formation up to 9 months on the other end, hence requiring subsequent procedure in the form of phemister bone grafting. Patients were followed for a minimum period of 2 years (range, 2–7 years). Results were based on the status of union and range of motion (ROM) for elbow/wrist and grip strength at the final follow-up. Complications observed were the reactivation of infection (n = 1) and herniation of the muscles at the donor site (n = 1).

**Conclusion:**

The tricorticocancellous strut bone grafting under optimal compression, augmented with intramedullary fixation, provides a promising solution to difficult problem of an atrophic nonunion of forearms bones with gap.

## INTRODUCTION

The management of a nonunion with a gap following a fracture of both bones of the forearm, both aseptic and septic, is a challenging problem. Nonunion is often the result of high-energy trauma with a massive loss of tissue, gunshot wounds, following resection of primary or secondary bone tumors, healed osteomyelitis with gap nonunion, or a complication of complex fractures with failed primary fixation.

Various treatment modalities described in treating forearm bone loss include using boiled cadavaric bones,^1^ use of cortical tibial graft with screws,^2^ grafts using ulnar segment held by screws,^3^ use of iliac crest graft to fill the bone gap and fixation with an intramedullary nail,^4^ cancellous insert grafts with plate fixation,^5^,^6^ bone transport in forearm bones using the principles of Ilizarov,^7^ using fibula as an intercalary bone graft and with a tibial cortical bone graft fixed opposite to a plate,^5^,^8^ and vascularized fibular iliac bone graft.^9^

The salvage procedures such as centralization of one bone as a treatment of segmental bone defect in forearm bones is also described.^10^,^11^

Davey^12^ modified Nicoll's technique and used blocks of the corticocancellous bone with a single cortex from the iliac crest, augmented with rigid plate fixation under compression. Ring^13^ used nonstructured autogeneous cancellous bone graft with plate fixation in patients with a diaphyseal nonunion of radius and/or ulna and reported good results when the soft tissue envelope is compliant having limited scars, and consists largely of healthy muscles with a good vascular supply. Edgardo^14^ reported good results using an Iliac crest graft with medullary nail fixation using a hun (hunec) nail.

The present paper discusses results of 38 forearm bones with segmental bone defects treated by tricorticocancellous bone graft under optimum compression with intramedullary fixation.

## MATERIALS AND METHODS

This prospective study was conducted between June 1985 and June 2005. Thirty-eight forearm bones in 23 patients of an atrophic nonunion with a gap regardless of age, sex, or prior surgical procedures but with some useful residual functions of wrist and fingers were included in the study. Cases with a preoperative active infection, no useful residual function of hand or those who could not be followed up to 2 years were excluded from the study.

The mean intercalary defect was 4.7 cm (range, 1.5–7.5 cm) after debridement. Two patients had the involvement of the median nerve and one patient had the involvement of the radial nerve preoperatively. Of the patients, 15 were males and 8 were females with a mean age of 44 years. Fifteen patients had fractures of both bones of the forearm, five had isolated fracture of the ulna and three had isolated fracture of the radius. Eighteen patients had been subjected up to one to three or less surgical procedures, and five cases were earlier treated conservatively.

### Operative procedure

The fracture site was exposed through standard surgical incisions. All the surrounding scarred tissue and sclerosed bone ends were excised till the fresh, visible blood-oozing surfaces were seen. A gentle traction and countertraction was given to measure the gap between the bony fragments. A tricorticocancellous iliac crest bone block, 2–3 mm longer than the measured gap, was harvested. The width of the graft was 2–2.5 cm. Some cancellous bone was also harvested from the remaining edges of the iliac crest. A longitudinal hole was drilled in the corticocancellious bone block, obtained with the help of a bone awl/drill (diameter of the hole was made greater than the thickness of the nail to be used). A square nail of predetermined size was negotiated in one of the fragments and was protruded through the fracture surface. The prepared strut (corticocancellous bone block) was threaded over the nail and rotated, as per local geometry of the bone and while maintaining sufficient traction, the strut was reduced between the fragments and the nail was driven further across the strut into the other fragment. Traction was then left which locked the corticocancellous block under optimum compression over a nail between the two fragments. Osteoperiosteal flaps were then raised on both bone host graft junctions and cancellous chips were impacted all around. The wounds were closed in layers over the suction drainage and the limb was immobilized in an elbow plaster of paris (POP) cast. The patients were encouraged to do active finger and shoulder exercises and were followed at 6-week interval, clinically and radiologically, till the fracture united radiologically.

**Figure 1:**
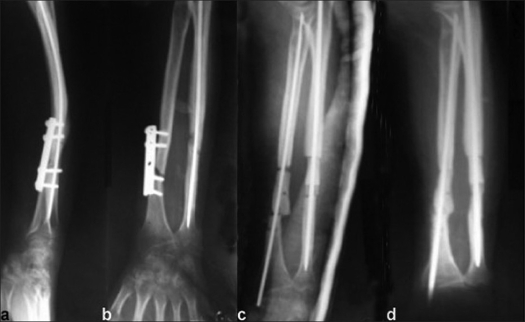
X-ray of right forearm lateral (a) and anteroposterior (AP) view (b) of a case of 10-month old ununited fracture both bones of the forearm with implant *in situ* (plate). AP view (c) shows fracture fixation with intramedullary nail and interposed tricorticocancellous strut bone graft. 20 weeks follow-up AP X-rays (d) shows fracture union with incorporation of bone graft

**Figure 2:**
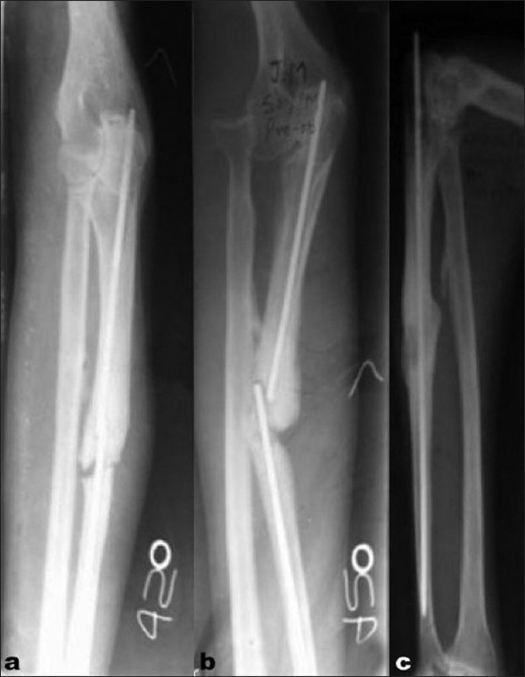
X-ray of left forearm with elbow lateral (a) and anteroposterior view (b) of a case of 2-year-old neglected Monteggia fracture dislocation with ununited ulna and broken implant *in situ* (intramedullary nail). Lateral view (c) shows radial head excision and fracture fixation with intramedullary nail and interposed tricorticocancellous strut bone graft incorporation at 24 weeks follow-up

Thus the authors have modified the Nicoll technique and used tricorticocancellous bone strut, to fill the bone gap, instead of wholly cancellous inserts used by Nicoll [Figures 1 and 2]. Nicoll removed the superior cortical plate of the iliac crest as “lid” and thin “wafer-like” cortical plates from both surfaces of the ilium and retained this wholly cancellous insert in the gap with a bridge plate without fixing the graft. We have used a part of the full iliac crest without removing any cortex, and stabilized the strut at the nonunion site by an intramedullary nail like a “signet” ring (graft with a hole in its whole length for the square nail).

## RESULTS

The patients were followed for a minimum of 2 years (range, 2–7 years). The mean duration of the nonunion was 24.8 months (range, 9–84 months).

Thirty-six bones showed union at both host graft junctions. The mean duration of the union was 17.5 weeks (range, 14–60 weeks). Two bones (case no. 5 and 14) had union only at one host graft junction and did not show any evidence of callus formation up to 9 months on other end, hence requiring a subsequent procedure in the form of phemister bone grafting to achieve union.

One case (case no. 10) had reactivation of infection but the fracture united at both the ends, taking 56 weeks to unite. The infection subsided after the removal of the implant by a standard procedure. Six cases had a poor grip strength in comparison to the grip strength of the opposite hand as measured by a dynamometer and three cases had stiffness of the wrist and fingers. One case had herniation of muscles at the donor site. Ten cases had full (>150°, mean 155°), five cases had 80 to 150° (mean115°), and rest eight cases had <80° (mean 65°) range of supination/pronation movement. But the range of motion at elbow, wrist, supination/pronation, fingers and grip strength improved in all the patients from their preoperative level. None of the cases had further neurovascular complications or breakage of implant or graft. The observations made have been tabulated in Table 1.

**Table 1:** Clinical details of patients

Pt. no.	Sex	Age	BI	PO	CI	DU (m)	Gap (cm)	UT (weeks)	Res.	Elbow		Wrist		Sup.	Pro.	GS	Compl. Add. pr.
												
										Flex.	Ext.	Df	Pf				
1	M	36	U	1	Comp. f.	18	5	18	Healed B.	F	F	F	F	F	F	F	–
2	M	32	R	2		16	5	16	Healed B.	F	-20	F	F	75%	75%	F	–
			U		Closed		5	20									
3	F	50	R	3		24	6	26	Healed B.	F	F	F	F	75%	75%	Poor	Stif. fin.
			U		Closed		5	26									
4	M	60	R	–	Comp. f.	26	3	24	Healed B.	F	F	F	F	F	F	F	–
			U				2										
5	M	28	R	2	Comp. f.	14	6	52	Healed O.	-10	-20	F	F	50%	75%	F	Phem.
			U				5	18									
6	F	50	U	–	Closed	28	3	26	Healed B.	F	F	F	F	F	F	F	–
7	F	65	R	1	Comp. f.	16	7	26	Healed B.	-20	-30	-10	F	50%	75%	F	–
8	M	25	R	1	Comp. f.	10	7.5	35	Healed B.	F	F	F	F	F	F	F	–
			U				4										
9	M	45	R	–	Comp. f.	40	5	23	Healed B.	F	F	-10	F	75%	75%	F	–
			U				4										
10	M	62	R	3	Comp. f.	36	6	56	Healed B.	F	F	-20	-20	50%	75%	Poor	Low infec.
			U				5										
11	M	42	R	1	Closed	9	1.5	14	Healed B.	F	F	F	F	F	F	F	–
12	M	37	R	3	Comp. f.	20	6	30	Healed B.	F	F	F	F	75%	F	F	–
			U				6										
13	F	30	U	1	Closed	15	4	14	Healed B.	F	F	F	F	F	F	F	–
14	F	67	R	2	Comp. f.	30	5	58	Healed O.	-20	-30	Nil	-10	50%	50%	Poor	Stif. fin., med. ner.
			U				6										phem.
15	M	42	R	3	Comp. f.	30	6	23	Healed B.	F	F	F	F	75%	75%	F	–
			U				5										
16	M	41	R	1	Comp. f.	9	2	16	Healed B.	F	F	F	F	F	F	F	–
			U				1.5										
17	M	30	R	2	Comp. f.	16	3	26	Healed B.	F	F	-10	-20	50%	75%	F	–
			U				4										
18	F	65	R	3	Comp. f.	40	6	60	Healed B.	-10	-20	Nil	-10	50%	75%	Poor	Hern., rad. ner.
			U				5										
19	F	30	R	1		10	2	14	Healed B.	F	F	F	F	F	F	F	–
			U		Closed		4										
20	F	45	R	–		84	6	48	Healed B.	F	-30	-10	-5	50%	75%	Poor	Stif. fin.
			U		Closed		7										
21	M	36	R	2	Comp. f.	32	7	14	Healed B.	F	F	F	F	F	F	F	–
22	M	20	U	2	Comp. f.	10	4	26	Healed B.	F	F	F	F	F	F	F	–
23	M	68	U	–	Comp. f.	36	6	28	Healed B.	-10	-20	-20	-10	75%	50%	Poor	Med. ner.

Pt. no.: patient's serial no., BI: bone involved, PO: no. of previous operations, CI: closed/compound fracture initially, duration (months), Gap: gap at fracture ends after debridement in cm, UT: union time, Res.: results, Flex: flexion, Ext.: extension, Df: dorsiflexion, Pf: palmarflexion, Sup.: supination, Pro.: pronation, GS: grip strength, Compl.: complications, Add. pr.: additional procedure required, U: ulna, R: radius, F: full, Comp. f.: compound fracture, Infec.: infection, Stif. fing.: stiff fingers, Phem.: phemister bone grafting, Med. ner.: median nerve, Rad. ner.,: radial nerve, Hern: herniation of muscles at the donor site, Healed B.: healed at both graft host junctions, Healed O.: healed at one graft host junction.

## DISCUSSION

An atrophic nonunion with or without a gap is often characterized by scarring, sclerosis, and absorption of bone at fracture margins, leading to scarcity of blood supply and lack of osteogenic potential due to biological failure. Various methods have been described to treat nonunions but there is a paucity of the literature toward the management of atrophic nonunions of forearm bones with gaps. Treatment modalities described [Table 2] include the use of boiled cadaveric bone, cortical tibial graft held with screws, grafts using ulnar segments held by screws, use of iliac crest graft with an intramedullary nail, cancellous insert graft with plate, bone transport using the principles of Ilizarov, use of fibula as an intercalary graft, vascularized fibular bone graft, and single bone forearm. Though Nicoll described a method of overcoming this difficult problem, by filling the defect left after the excision of the dead bone, with cancellous bone from the anterior iliac crest and securing the graft by plate and screws, there are very few published reports on Nicoll's technique. This simple technique seems to have fallen in popularity, and is not being practiced by masses as a standard orthopedic procedure to overcome the challenging problem of atrophic nonunions of single or both bones of the forearm. The present study was undertaken to treat nonunions with a gap in forearm bones by modified the Nicoll technique.

**Table 2:** Results of various studies and there comparison with present study

Year	No. of bones/cases	Gap mean (range)	Union time mean (range)	Failure (secondary procedures)	Complications	Criteria, if any
Spira, 1954	14 bones in 13 cases	5 cm (About)	–	2	Infection-2	Bony union
Nicoll, 1956	14 forearm bones	(1-1^3/8^ inch)	14 weeks (6 weeks-6 months)	None	–	Bony union
Davey, 2002	19 patients	3.7 cm (2-6 cm)	13 months (3-24 months)	Non union-1 Sec.proce-2	–	Bony union
David and Ring, 2004	35 patients	2.2 cm (1-6 cm)	6 months	Unsatisf-11 Poor -1	Arthrosis of DRUJ-2 Chr. pain-2	Anderson
Edgardo, 2005	44 bones in 38 cases	5.2 cm (3-7.5 cm)	16.5 weeks (13-20 weeks)	4	Sepsis-2 Tendinitis-1 Pain at the donor site-2	Bony union
					
Present study	38 bones in 23 cases	4.7 cm (1.5-7.5 cm)	17.5 weeks (14-60 weeks)	2 (phemister at one host graft junction)	Herniation of muscles-2 Low grade infection-1	Bony union

Nonvascularized autografts are the most commonly used grafts and are considered to be the “gold standard.” They have osteoconductive and osteoinductive properties as well as osteoprogenitor cells.^15^,^16^ It is clear both from the clinical and experimental evidence that a fresh autogenous cancellous bone is far superior to any other form of transplant. For physical reasons, solid blocks give a more rapid union and less fibroblastic reactions than chips or ribbons.^5^,^15^ Host tissues into which the transplant is made must have a good blood supply, since osteogenesis, whether by survival, invasion, or induction, can occur only if the graft is in intimate contact with an active circulation. All sclerotic bone ends must therefore be removed, even though this increases the gap. These sclerotic ends are devoid of the blood supply and are quite incapable of providing an outflow of the osteogenetic tissue into the graft. This outflow from the host bone is regarded by Siffert^17^ as the most important single source of osteogenesis, so the surgeon must not be afraid of cutting back to a normal healthy vascular bone. The gap may look formidable at operation but a 2-inch defect between healthy fragments is more likely to be bridged than a 1-inch defect between fibrotic fragments.^5^ The muscle bed must also be freshened by the removal of avascular fibrous tissue because osteogenesis by induction can occur only if a cancellous surface is presented to a vascular muscle bed, as in the experimental transplants of Urist and McLean.^18^ The stabilization of a nonunion site also helps in improving the function and range of motion. David^13^ also reported a better range of motion than before surgery in all his patients, even in those who had been injured 3–10 years before surgery and had undergone several previous operations.

This technique of grafting is simple; it provides stability to fracture as well as bone graft. The presence of cortical bones on three sides provides optimal compression at both host graft junction without crushing/fracture of wholly cancellous block and enough strength till it is incorporated. The thin cortical bone on the two sides allows for early invasion of the periosteal circulation.^14^ Abundance of the cancellous bone decreases the time of graft incorporation and gaps in early healing of the fracture. Thus, the procedure serves in three ways, i.e., internal fixation, defect bridging, and osteogenesis promoter. The intramedullary nail gives a stable fixation to the fracture and the graft without weakening of the graft. The removal of the nail is a minor procedure and does not require exposure of the grafted area. Whereas removal of plate and screws require major surgery if so required. Moreover, autografts do not have any risk of immunological response or transfer of disease, or the risk of pin tract infection and do not require a painful heavy ring fixator for bone transport. Functionally, all the patients had a better range of motion and grip strength, than before surgery, even in those who had been injured 7 years before surgery or had undergone multiple surgical interventions prior to this final surgical intervention. The only specific complication is the risk of herniation of muscles at the donor site, which is amicable to treatment. Thus, this simple technique of tricorticocancellous bone grafting under optimal compression, augmented with intramedullary fixation, provides a promising solution to this difficult problem of atrophic nonunion with a gap. This technique is simple, can be done by any orthopedic surgeon without modern sophisticated instrumentation yielding promising results.
